# Impact of therapeutic hypothermia on infantile spasms: an observational cohort study

**DOI:** 10.1111/dmcn.14331

**Published:** 2019-09-13

**Authors:** Farah Abu Dhais, Brian McNamara, Olivia O'Mahony, Niamh McSweeney, Vicki Livingstone, Deirdre M Murray, Geraldine B Boylan

**Affiliations:** ^1^ INFANT Research Centre, University College Cork Cork Ireland; ^2^ Department of Paediatrics and Child Health University College Cork Cork Ireland; ^3^ Department of Neurophysiology Cork University Hospital Cork Ireland; ^4^ Department of Paediatrics Cork University Hospital Cork Ireland

## Abstract

**Aim:**

To establish the incidence of infantile spasms in children in the southern region of the Republic of Ireland and to compare the incidence of infantile spasms before and after the introduction of therapeutic hypothermia in infants with hypoxic‐ischemic encephalopathy (HIE).

**Method:**

Children born between 2003 and 2015 and diagnosed with infantile spasms (epileptic spasms with or without hypsarrhythmia) in the first 2 years of life were identified through audits of electroencephalography reports and paediatric neurology patient lists. Data on live births were obtained from the regional hospital statistics databases. Medical charts of infantile spasm cases were reviewed for demographic information, diagnostic workup results, treatment response, disease course, and developmental outcome.

**Results:**

Forty‐two infants with infantile spasms were identified. The cumulative incidence of infantile spasms up to the age of 2 years was 4.01 per 10 000 live births. Difference due to sex was minimal (22 males, 20 females) and most infants were delivered at or near term with gestational ages ranging between 30.0 and 41.8 weeks (median [interquartile range] 39.6wks [38.1–40.0wks]). The aetiology for infantile spasms was identified in almost two‐thirds of cases, with HIE being the single most common cause (*n*=7). Other causes included chromosomal and monogenetic abnormalities (*n*=8). Infantile spasms occurred in moderate and severe grades of HIE, with a significantly higher incidence in those with severe HIE (*p*=0.029). Infants with severe HIE who did not receive therapeutic hypothermia were six times more likely to develop infantile spasms compared to those who did, but the difference was not statistically significant (4 out of 16 vs 1 out of 24, *p*=0.138).

**Interpretation:**

This study provides detailed information about infantile spasms before and after the introduction of therapeutic hypothermia. HIE severity is a risk factor for the development of infantile spasms. The introduction of therapeutic hypothermia may have had an impact, but the effect was hard to ascertain in this cohort due to the small number of infants.

**What this paper adds:**

- The incidence of infantile spasms and patient characteristics in the southern region of the Republic of Ireland is similar to internationally published data.
- None of the infants with a history of mild hypoxic‐ischemic encephalopathy (HIE) developed infantile spasms.
- The risk of infantile spasms was higher in infants with severe HIE.
- Infantile spasms were more frequent in infants with severe HIE not treated with therapeutic hypothermia.

## What this paper adds

- The incidence of infantile spasms and patient characteristics in the southern region of the Republic of Ireland is similar to internationally published data.
- None of the infants with a history of mild hypoxic‐ischemic encephalopathy (HIE) developed infantile spasms.
- The risk of infantile spasms was higher in infants with severe HIE.
- Infantile spasms were more frequent in infants with severe HIE not treated with therapeutic hypothermia.

This article's abstract has been translated into Spanish and Portuguese.

Follow the links from the http://onlinelibrary.wiley.com/doi/10.1111/dmcn.14331/abstract to view the translations.

This article is commented on by Vollmer on page https://doi.org/10.1111/dmcn.14386 of this issue.

## Resumen

**Objetivo:**

Establecer la incidencia de los espasmos infantiles en niños de la República de Irlanda y comparar la incidencia de los espasmos infantiles antes y después de la introducción de la hipotermia terapéutica en niños con encefalopatía hipóxico‐isquémica (EHI).

**Método:**

Niños nacidos entre 2003 y 2015 y diagnosticados con espasmos infantiles (espasmos epilépticos con o sin hipsarritmia) en los primeros 2 años de vida fueron identificados por medio de auditorías de reportes de electroencefalográficos y listas de pacientes de neurólogos infantiles. Datos sobre los nacidos vivos se obtuvieron de la base de datos estadística del hospital regional. Las historias clínicas de los casos de espasmos infantiles fueron revisadas para obtener datos demográficos, resultados diagnósticos, respuesta a tratamiento, curso de la enfermedad y resultados del desarrollo.

**Resultados:**

Fueron identificados 42 niños con espasmos infantiles. La incidencia acumulada de los espasmos infantiles por encima de los 2 años fue de 4,01 por 10.000 nacidos vivos. La diferencia debida al sexo fue mínima (22 masculinos, 20 femeninos) y la mayoría nacieron en o cercano a término, con edad gestacional entre 30,0 y 41,8 semanas (media (rango Intercuartil) 39,6 semanas (38,1‐40,0 semanas). La etiología de espasmos infantiles fue identificada en dos tercios de los casos, siendo EHI la causa más común (7 de 42). Otras causas incluyeron anormalidades cromosómicas y monogénicas (8 de 42). Los espasmos infantiles ocurrieron en los grados moderados y severos de EHI con incidencia significativamente mayor en aquellos casos severos de EHI (p=0,029). Los niños con EHI severo que no recibieron hipotermia terapéutica tuvieron una probabilidad seis veces mayor de desarrollar espasmos infantiles comparados con aquellos quienes la recibieron, pero la diferencia no era estadísticamente significativa (4 de 16 vs 1 de 14, p=0,138).

**Interpretación:**

Este estudio proporciona información detallada acerca de los espasmos infantiles antes y después de la introducción de la hipotermia terapéutica. La severidad de la EHI es un factor de riesgo para el desarrollo de los espasmos infantiles. La introducción de la hipotermia terapéutica puede haber tenido un impacto, pero el efecto fue difícil de determinar en esta cohorte debido al pequeño número de recién nacidos.

## Resumo

**Objetivo:**

Estabelecer a incidência de espasmos infantis em crianças da República da Irlanda e comparar a incidência de espasmos infantis antes e após a introdução da hipotermia terapêutica em lactentes com encefalopatia hipóxica‐isquêmica (EHI).

**Método:**

Crianças nascidas entre 2003 e 2015 e diagnosticadas com espasmos infantis (espasmos epilépticos com ou sem hipsarritmia) nos primeiros 2 anos de vida foram identificadas por meio de checagem dos relatórios de eletroencefalografia e listas de pacientes de neurologia pediátrica. Dados sobre nascidos vivos foram obtidos nas bases de dados estatísticas dos hospitais regionais. Prontuários médicos sobre casos de espasmos infantis foram revisados quanto a dados demográficos, resultados diagnósticos, resposta ao tratamento, curso da doença, e resultado desenvolvimental.

**Resultados:**

Quarenta e dois lactentes com espasmos infantis foram identificados. A incidência cumulativa de espasmos infantis até os dois anos de idade foi 4,01 por 10.000 nascidos vidos. Diferenças devida ao sexo foram mínimas (22 meninos, 20 meninas) e a maior parte dos lactentes nasceu próximo ao termo, com idades gestacionais variando de 30,0 41,8 semanas (mediana [intervalo interquartil] 39,6 sem [38,1–40,0sem]). A etiologia dos espasmos infantis foi identificada em dois terços dos casos, com a EHI sendo a causa mais comum (7 em cada 42). Outras causas incluíram anormalidades cromossômicas e monogenéticas (8 em 42). Os espasmos infantis aconteceram em graus moderados a severos de EHI, com incidência significantemente maior naqueles com EHI severa (*p*=0,029). Lactentes com EHI severa que não receberam hipotermia terapêutica tinham seis vezes mais probabilidade de desenvolver espasmos infantis comparados com os que receberam, mas a diferença não foi estatisticamente significativa. (4 em16 vs 1 em 24, *p*=0,138).

**Interpretação:**

Este esudo fornece informação detalhada sobre espasmos infantis antes e após a introdução de hipotermia terapêutica. A severidade da EHI é um fator de risco para o desenvolvimento de espasmos infantis. A introdução de hiportermia terapêutica por ter tido um impacto, mas o efeito foi difícil de assegurar nesta coorte devido ao pequeno número de lactentes.

**Abbreviation**
HIE: Hypoxic-ischemic encephalopathy

**What this paper adds**

- The incidence of infantile spasms and patient characteristics in the southern region of the Republic of Ireland is similar to internationally published data.
- None of the infants with a history of mild hypoxic‐ischemic encephalopathy (HIE) developed infantile spasms.
- The risk of infantile spasms was higher in infants with severe HIE.
- Infantile spasms were more frequent in infants with severe HIE not treated with therapeutic hypothermia.

Infantile spasms were first reported by Dr West in 1841.^1^ Historically, infantile spasms and West syndrome have often been used synonymously. Current established terminology describes West syndrome as a subset of infantile spasms, consisting of the combination of spasms and a hypsarrhythmic electroencephalogram (EEG) (Fig. S1, online supporting information). Often, but not necessarily, they are accompanied by developmental delay and/or regression.^2^, ^3^, ^4^ Infantile spasms are described as an age‐specific epileptic encephalopathy, with a clinically distinguished seizure type, which may or may not be accompanied by the characteristic EEG findings of hypsarrhythmia.^5^ Infantile spasms have an estimated incidence of 2 to 5 per 10 000 live births.^6^ Spasms typically present as clusters of sudden brief flexion, extension, or mixed extension–flexion contractions of proximal and truncal muscles, followed by a sustained tonic phase lasting up to 2 seconds.^3^, ^7^, ^8^ These often resolve over time, but up to 50% of patients develop other types of refractory epilepsy syndromes, such as Lennox–Gastaut syndrome.^9^

Infantile spasms are further categorized into two subgroups: infantile spasms with a known and clearly identifiable cause (formerly known as symptomatic), including metabolic, structural, and genetic causes; and infantile spasms of unknown cause.^10^, ^11^ A better prognosis is reported in the majority of patients in the latter subgroup.^5^, ^12^ Hypoxic‐ischemic encephalopathy (HIE) forms the major aetiology of infantile spasms,^13^ but limited research that evaluates the effect of therapeutic hypothermia on the incidence and outcome of infantile spasms has been published.^14^

Early diagnosis and treatment of infantile spasms are believed to improve outcome, especially if started within the first month of the onset of spasms.^12^ The aims of treatment are to obtain spasm cessation, normalize the EEG, and achieve better long‐term developmental outcomes. Treatment options consist of standard first‐line pharmacological agents, including hormonal therapy and vigabatrin, and non‐standard options including other medications, a ketogenic diet, and surgical intervention.^3^ Because of the rarity of the disorder, experimental trials are difficult to perform and are therefore limited.

In this study, we describe the overall incidence, causes, and disease outcomes of infantile spasms in infants born between January 2003 and December 2015 in the southern region of the Republic of Ireland.

Therapeutic hypothermia was introduced into neonatal intensive care across the Republic of Ireland in 2009. Therefore, for infants with moderate and severe HIE, we also examined any possible effect(s) of therapeutic hypothermia on the incidence of infantile spasms in infants with a history of HIE.

## Method

This was a retrospective observational study conducted at Cork University Hospital and Mercy University Hospital, the only two hospitals with paediatric neurology departments covering the southern territories of the Republic of Ireland. For the purposes of this study, the term ‘infantile spasms’ will be used to describe epileptic spasms characterized by sudden brief movements of the trunk and limbs, with a flexor, extensor, or mixed pattern developing in infants within the first 2 years of life, with or without the presence of classic hypsarrhythmia or its variants (modified hypsarrhythmia) on EEG.^5^, ^7^ Where applicable, patients diagnosed with infantile spasms were divided into those with hypsarrhythmia and those without, where classic hypsarrhythmia is represented by an interictal EEG with a chaotic high voltage background pattern intermixed with generalized and multifocal discharges, and modified hypsarrhythmia corresponds to hypsarrhythmia with atypical features (focal or asymmetrical, episodic attenuation, or interhemispheric synchronization).^15^, ^16^, ^17^

Children with infantile spasms were identified in two stages. First, through an audit of EEG reports from the neurophysiology departments of the two hospitals (Cork University Hospital and Mercy University Hospital) that provide services to the region. This was done by a computer search of EEG reports, extending from January 2003 to December 2017, using the following keywords: hypsarrhythmia, spasm, infantile, West, salaam/salam, and epileptic encephalopathy. All EEG recordings were reviewed and reported by a consultant clinical neurophysiologist (BM). The second stage of the audit was completed at the paediatric neurology departments at Cork University Hospital and Mercy University Hospital, whose staff are responsible for the follow‐up of all children with new‐onset seizures in the region. Paediatric neurologists (NM, OO) and clinical nurse specialists in epilepsy identified additional patients missed in the first stage of our search. Children born in or admitted to any of the Cork maternity hospitals with tertiary neonatal units between January 2003 and December 2015, with a confirmed diagnosis of infantile spasms within 2 years of birth, were included in the study. (During the 2003 to 2007 period, there were two maternity hospitals with level 3 neonatal units covering Cork city: Erinville Hospital and St. Finbarr’s Maternity Hospital. After amalgamation in March 2007, Cork University Maternity Hospital became solely responsible for maternity and neonatal admissions in the area). Data regarding the number of live births in the region were available from 2006 onwards from Cork University Hospital Perinatal Statistics (2005– 2016). Thus, for the purpose of calculating cumulative incidence, children born after 2005 and diagnosed with infantile spasms were included.

Next, an audit review of the medical charts of infants diagnosed with infantile spasms was completed and the following data were collected: demographics including date of birth, sex, birthweight, gestational age, neonatal course, and family history of seizure disorder or developmental delay; initial investigations including EEG reports; and aetiological testing results. Aetiological tests included brain imaging, and genetic and metabolic workup. Metabolic workup was individualized and tailored to each patient. Investigations included lactate, ammonia, amino acids, creatine kinase, urate, urinary purines and pyrimidines, urinary organic acids, biotinidase, mucopolysaccharides, acylcarnitine, lysosomal enzymes and very‐long‐chain fatty acids, and cerebrospinal fluid neurotransmitters. Genetic testing in the earlier period of the study comprised karyotyping; later on, comparative genomic hybridization microarray was introduced. In more recent years (2010 onwards), early epileptic encephalopathy gene panels have been used. Depending on the results of aetiological testing, patients were divided into infantile spasms of known aetiology and infantile spasms of unknown aetiology. This study was approved by the Clinical Research Ethics Committee of the Cork Teaching Hospitals. 

### Treatment response

Treatments were decided by the attending paediatric neurologist, taking into account associated comorbidities and parental preferences; response to treatment was assessed within 2 weeks of initiation. Infants were classified into two groups based on their response: non‐responders, if there was minimal to no effect after 2 weeks of treatment; and responders, if there was resolution of both the spasms and the electrographic abnormalities persisting for longer than 6 weeks. If no follow‐up EEG was reported, response was based on clinical cessation of spasms.

### Disease course including age of diagnosis and disease outcome

Disease outcome was described with regard to subsequent epilepsy, progression to Lennox–Gastaut syndrome, death, and developmental outcome. Lennox–Gastaut syndrome was defined as infants having multiple types of seizures (mainly tonic and atypical absence seizures), being unresponsive to treatment and having slow spike‐and‐wave discharges and paroxysmal generalized fast activity on EEG (Fig. S1), and intellectual delay.^18^, ^19^ Refractory epilepsy was used for infants with ongoing epilepsy (at the time of data collection) despite multiple treatment trials (three or more antiepileptic medications). Development was assessed by paediatric neurologists (NM, OO) on follow‐up, under the four major domains: gross motor; fine motor and vision; speech and hearing; and social skills. Infants were considered to have global developmental delay if two or more developmental domains were affected.

### Infants with HIE

During the period from 2003 to 2006, infants with a diagnosis of HIE were prospectively recruited at the Cork Teaching Hospitals, to research projects approved by the Clinical Research Ethics Committee of the Cork Teaching Hospitals, involving continuous EEG monitoring and long‐term follow‐up assessments of neonates at high risk of seizures.^20^ Between 2008 and 2015, the total number of infants admitted with HIE was obtained from the Cork University Maternity Hospital In‐Patient Enquiry scheme database (data were not available for 2007). Data including sex, gestational age, clinical grade of encephalopathy (Sarnat staging),^21^ and therapy received (therapeutic hypothermia vs no therapeutic hypothermia) were recorded for infants diagnosed during the aforementioned periods. From 2009 onwards, the decision to administer therapeutic hypothermia was based on Sarnat staging, with moderate and severe grades of HIE undergoing treatment as per the TOBY study.^22^ Access to neonatal clinical and research EEGs and postneonatal follow‐up EEGs carried out on an outpatient basis was available for the purposes of this review.

### Statistical analysis

Statistical analysis was performed with IBM SPSS Statistics version 24.0 (IBM Corp., Armonk, NY, USA). Cumulative incidence was calculated as the proportion of infants diagnosed with infantile spasms up to the age of 2 years among live births. A 95% confidence interval (CI) for the cumulative incidence was calculated using a binomial distribution (Clopper–Pearson exact method). For the descriptive analysis of normally distributed continuous variables, the mean and SD were used; for non‐normally distributed variables, the median and interquartile range (IQR) were used. Categorical variables were described using frequencies and percentages. Fisher’s exact test was used for the comparative analysis of categorical variables between groups; the Mann–Whitney *U* test was used for the comparative analysis of continuous, non‐normally distributed variables between groups. All tests were two sided and a *p*‐value less than 0.05 was considered to be statistically significant.

## Results

During the study period, 47 infants had a confirmed diagnosis of infantile spasms within 2 years of birth. Of those, five were born outside the study area and were excluded from the study. Thus, 42 infantile spasm cases were included in the study analysis. Thirteen infants developed different types of seizures before the onset of infantile spasms, including 10 who had seizures during the neonatal period (first 28d of life). The age at infantile spasm diagnosis ranged from 6 weeks to 21 months.

Of the 42 infants, 33 were born during the years 2006 to 2015, the years for which data regarding the number of live births were available. Accordingly, the cumulative incidence of infantile spasms up to the age of 2 years among live births was 4.01 per 10 000 (95% CI=2.76–5.63). The yearly cumulative incidence varied, with the highest occurring in 2013, reaching 8.43 per 10 000 (Table 1).

**Table 1:** Yearly cumulative incidence of infantile spasms up to the age of 2y among live births

Year	Total births^a^	Stillbirths	Live births (excluding stillbirths)	Infantile spasm cases	Cumulative incidence (per 10 000)
2006	6090	24	6066	3	4.95
2007	7998	39	7959	1	1.26
2008	8778	43	8735	2	2.29
2009	8978	44	8934	1	1.12
2010	8898	43	8855	2	2.26
2011	8786	30	8756	6	6.85
2012	8563	32	8531	3	3.52
2013	8339	32	8307	7	8.43
2014	8071	39	8032	3	3.74
2015	8113	38	8075	5	6.19
Total	82 614	364	82 250	33	4.01 (2.76–5.63)^b^

a Inclusive of stillbirths and neonatal deaths.

b 95% confidence interval.

### Demographics

The number of males and females was similar (Table 2). Twenty‐four infants were delivered by vaginal delivery and 18 by Caesarean section. Gestational age varied between 30.0 and 41.8 weeks. Only 5 infants were delivered preterm (<37wks), including a set of twins. Fourteen infants had a positive family history of epilepsy, four had a family member with febrile convulsions, and eight had a family relative with developmental delay or learning difficulties.

**Table 2:** Characteristics of patients diagnosed with infantile spasms

	Total (*n*=42)	Infantile spasms of unknown aetiology (*n*=15)	Infantile spasms of known aetiology (*n*=27)	*p* a
Sex (male), *n* (%)	22 (52.2)	12 (80.0)	10 (37.0)	0.011
Birthweight (kg), median (IQR)	3.5 (2.9–3.7)	3.5 (3.2–3.7)	3.4 (2.9–3.7)	0.844^b^
Gestational age (wk), median (IQR)	39.6 (38.1–40.0)	38.7 (37.0–40.0)	39.7 (38.6–40.0)	0.323^b^
Neonatal intensive care unit admission, *n* (%)	24 (57.1)	5^c^ (33.3)	19^d^ (70)	0.027
Infantile spasm age of diagnosis (mo), median (IQR)	7.0 (5.0–11.0)	8.0 (6.0–11.0)	7.0 (5.0–10.0)	0.257^b^
Hypsarrhythmia, *n* (%)	38 (90.5)	12^e^ (80.0)	26^f^ (96.3)	0.122
Duration of follow‐up (mo), median (IQR)	61.2 (22.6–84.2)	44.3 (23.0–67.4)	63.4 (20.0–90.2)	0.684^b^
Epilepsy post‐infantile spasms, *n* (%)	28 (66.7)	8 (53.3)	20 (74.1)	0.193
Lennox–Gastaut syndrome, *n* (%)	6 (14.3)	2 (13.3)	4 (14.8)	1
Global developmental delay, *n* (%)	35 (83.3)	10 (66.7)	25 (92.6)	0.077
Final outcome	–	–	–	0.016
Resolved infantile spasms/epilepsy, *n* (%)	19 (45.2)	11 (73.3)	8 (29.6)	0.010
Resolved epilepsy, *n* (%)	11 (26.2)	3 (20.0)	8 (29.6)	0.717
Death, *n* (%)	12 (28.6)	1 (6.7)	11 (40.7)	0.031

a Fisher’s exact test, unless otherwise stated.

b Mann–Whitney *U* test.

c Causes of admission: poor feeding (*n*=1); jaundice (*n*=1); preterm birth (*n*=2); non‐clinical reasons, maternal cardiomyopathy requiring stay in intensive care (*n*=1).

d Causes of admission: respiratory distress (*n*=6); hypoxic‐ischemic encephalopathy (*n*=7); perinatal asphyxia (*n*=1); sepsis (*n*=1); poor feeding (*n*=1); preterm birth (*n*=1); bloody stool for further investigations (*n*=1); antenatally diagnosed cardiac lesions for echocardiography (*n*=1).

e Of the 12 infants, seven had classical hypsarrhythmia and five had modified hypsarrhythmia.

f Of the 26 infants, 21 had classical hypsarrhythmia and five had modified hypsarrhythmia. IQR, interquartile range.

### Neonatal course

Over half of the infants (*n*=24) were admitted to the neonatal unit after delivery; HIE was the most common reason for admission (*n*=7; Table 2).

Neonatal EEGs were recorded in 12 infants: 10 were initiated for clinical purposes (and later enrolled in research projects) and two as part of a research project only. All 10 infants who had a clinically indicated EEG, had suspected seizures for which they were treated. Among those, electrographic seizures were documented in six.

### Aetiology of infantile spasms

All infants were investigated to determine a possible aetiology. Forty‐one patients had a brain MRI and one had a computed tomography scan performed. Metabolic testing and karyotyping/comparative genomic hybridization microarray were performed for all patients who had no apparent abnormality on brain imaging; an epilepsy gene panel was sent for five patients.

Based on aetiological testing, 27 infants had a known aetiology (Table 2). Various causes were identified, with HIE being the most predominant (*n*=7), followed by trisomy 21 and brain malformations (*n*=5 each). Brain malformations included lissencephaly, polymicrogyria, Miller–Dieker syndrome, Aicardi syndrome, and holoprosencephaly. Three infants were diagnosed with tuberous sclerosis and three others had genetic epileptic encephalopathies identified (1p36 deletion, 15q duplication, and CDKL5 mutation). Less frequent aetiologies were periventricular leukomalacia, brain tumour (left temporoparietal desmoplastic infantile ganglioglioma), neonatal stroke (left middle cerebral artery territory), and neonatal meningitis (group B *Streptococcus*), (*n*=1 each).

### EEG findings at the time of infantile spasms

Thirty‐eight infants had hypsarrhythmia (28 classical and 10 modified; Table 2). The remaining four infants diagnosed with infantile spasms presented with clinical events and abnormal EEGs but no hypsarrhythmia. They were divided as follows: in the group of infantile spasms of unknown aetiology (*n*=15), three had an epileptic EEG but no hypsarrhythmia; in the group of infantile spasms of known aetiology (*n*=27), only one had a non‐hypsarrhythmic EEG. The latter infant had a history of HIE and had received therapeutic hypothermia; his EEG was diffusely encephalopathic.

### Pharmacological therapy

The most common first‐line treatment was vigabatrin (*n*=25), followed by prednisolone (*n*=15), both dosed according to the United Kingdom Infantile Spasms Study protocol.^23^ The response rates for vigabatrin and prednisolone were 8 out of 25 and 5 out of 15 respectively. Two infants (one from each group) were switched to a different medication due to side effects. Other first‐line treatments were sodium valproate given to an infant with trisomy 21, and levetiracetam prescribed for focal seizures coexisting with spasms, before starting vigabatrin (*n*=1 each).

One‐third of infants (*n*=13) responded to the first‐line medication. Three infants had no records of follow‐up EEG after their infantile spasm diagnosis, two died after redirection of care, and one child moved to another neurology centre outside the region. All three had no clinical response to their first‐line medication.

Second‐ and third‐line medications included those from first‐line choices, as well as adrenocorticotropic hormone, clonazepam, topiramate, and clobazam. Fifteen infants received pyridoxine as part of their treatment protocol; 19 infants required three or more medications during the course of their illness.

### Disease outcome

Infants were followed up for a median (IQR) of 61.2 months (22.6–84.2mo) from the time they were diagnosed until the end of the study period or the date of death (where applicable). Two‐thirds (*n*=28) developed other forms of epilepsy after infantile spasms. Six were diagnosed with Lennox–Gastaut syndrome. The remainder (*n*=22) developed focal (*n*=4), typical/atypical absence (*n*=2), tonic (*n*=2), atonic (*n*=1), generalized tonic–clonic (*n*=3), and myoclonic seizures (*n*=5), and combinations of two or more of the aforementioned types of seizures (*n*=5).

Regarding development, 35 infants were globally delayed, including 12 infants diagnosed with cerebral palsy, 11 had spastic quadriplegic cerebral palsy, and 1 had hemiplegic cerebral palsy. Comorbidities included visual impairment (*n*=9), microcephaly (*n*=8), autism (*n*=4), and sensorineural hearing loss (*n*=2). Seven infants did not have global developmental delay; of these, four had language delay and attended speech and language therapy. The percentage of infants with global developmental delay was higher in the infantile spasms of known aetiology group compared to the infantile spasms of unknown aetiology group, but the difference was not statistically significant (25 out of 27 vs 10 out of 15, *p*=0.077).

At the time of the study, 12 infants had died between the ages of 6 months and 10 years (median [IQR]:  24.5mo [21.0–70.7mo]) and this percentage was higher in the infantile spasms of known aetiology group compared to the infantile spasms of unknown aetiology group (11 out of 27 vs 1 out of 15, *p*=0.031). Of the surviving infants (*n*=30), 19 had resolved spasms/epilepsy and 11 had refractory epilepsy.

### Infantile spasms secondary to HIE

A total of 178 infants with HIE were admitted to the neonatal units during the 2003 to 2006 and 2008 to 2015 periods (74 females, 104 males). Their clinical HIE grades were divided as follows: 49 mild; 89 moderate; and 40 severe. Seventy‐eight out of 178 received therapeutic hypothermia.

Among the 178 infants with HIE, seven developed infantile spasms (four males, three females), giving a cumulative incidence of 3.93% (95% CI=1.60–7.93). The infants with infantile spasms secondary to HIE (*n*=7) had neonatal EEG recordings consistent with severe encephalopathy (burst suppression pattern and/or isoelectric trace; Fig. S1). All seven infants developed clinical seizures and five had documented electrographic seizures during their neonatal intensive care unit stay. The remaining two infants with no recorded electrographic seizures had already received antiepileptic drugs before the start of EEG monitoring. Brain MRI revealed marked abnormality in the parietal lobes and basal ganglia in five infants, and extensive bilateral injury to the cortical and subcortical areas and deep grey matter involvement in two infants.

**Figure 1:**
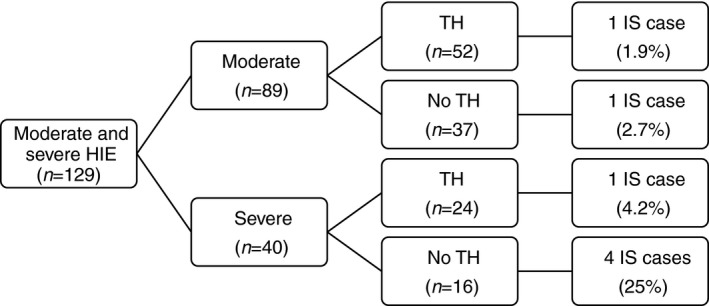
Percentages of infantile spasms (IS) among moderate and severe hypoxic‐ischemic encephalopathy (HIE) in Cork (2003–2006 and 2008–2015). Flow chart of infants with moderate and severe HIE showing the highest percentage of later infantile spasm development in the severe/no therapeutic hypothermia (TH) subgroup.

The seven infants with later infantile spasms had moderate‐to‐severe Sarnat staging of HIE as neonates, with the degree of severity significantly increasing the risk of infantile spasm development. Of the 40 infants with severe HIE, five developed infantile spasms; of the 89 infants with moderate HIE, only two developed infantile spasms (12.5% vs 2.2%, *p*=0.029). Taking into consideration that therapeutic hypothermia may affect the outcome, we looked at untreated infants only and compared the incidence of infantile spasms between the severe and moderate groups; a statistically significant difference remained between the two groups (4 out of 16 vs 1 out of 37, *p*=0.025; Fig. 1).

In the infants with severe HIE, 24 received therapeutic hypothermia and 16 did not (12 were born before its introduction and four were not treated because of preterm birth or delayed diagnosis/transfer).  Infants with severe HIE who did not receive therapeutic hypothermia were six times more likely to develop infantile spasms compared to those who did, but the difference was not statistically significant (4 out of 16 vs 1 out of 24, *p*=0.138; relative risk [95% CI]: 0.17 [0.02–1.36]). For infants with moderate HIE, the incidence of infantile spasms did not differ between untreated and treated infants (1 out of 37 vs 1 out of 52, *p*=1).Of the seven infants with infantile spasms secondary to HIE, the five infants who did not receive therapeutic hypothermia died by the age of 7 years, while the two treated infants survived but had global developmental delay and epilepsy.

## Discussion

We have shown that the cumulative incidence rate of infantile spasms in the southern region of the Republic of Ireland is 4.01 per 10 000 live births. HIE continues to be a major cause of infantile spasms, with the severity of HIE staging linked to the incidence of infantile spasms as a long‐term outcome. Outcome and response to treatment remain poor in patients with infantile spasms, with nearly a third dying before the age of 10 years.

Results from our study showed cumulative incidence rates, sex, age at which spasms were diagnosed, and developmental outcomes that are comparable to previously published data.^6^, ^9^, ^12^, ^24^, ^25^, ^26^ Infantile spasms are more commonly seen in infants born at term than in infants born preterm, possibly reflecting the effect of brain maturation on the risk of spasm development. Prior studies documented that infants born preterm formed only 5% of the total numbers of infantile spasm patients,^13^, ^27^ whereas our cohort had a relatively higher percentage of 12%.

A clear aetiology was identified in almost two‐thirds of infants, as is the case in similar observational cohorts.^13^, ^24^ In the United Kingdom Infantile Spasms Study, HIE was reported as the most common cause of infantile spasms, accounting for 10% of all aetiologies.^13^ Likewise, HIE was observed to be the most common cause of infantile spasms in our group. Trisomy 21 formed another major subset, which could be explained by the high prevalence rate of trisomy 21 in Ireland, recently estimated at 35.7 out of 10 000 births.^28^ In our cohort, only five infants had an epilepsy gene panel investigated, which could explain the limited number of documented genetic mutations reported in this study. Nowadays, epilepsy gene panels form an integral part of the investigative process. Furthermore, whole‐genome sequencing has been explored for possible use in patients who have had negative genetic diagnosis through early‐onset epileptic encephalopathy panels and has shown promising results.^29^

In the group under study, almost all infants were started on either vigabatrin or steroids after diagnosis, and similar response rates were found for both, which is contradictory to previous evidence of better response with hormonal treatment.^23^ Recent reports indicate that dual therapy (vigabatrin and steroids) is more effective than hormonal therapy alone, having a higher rate of spasm cessation and a shorter response time.^30^ Impact on neurodevelopment with this therapeutic approach will need further exploration.

Poor long‐term outcomes in infants with infantile spasms are well documented, with a large proportion progressing to other seizure types, often resistant to treatment.^3^ Additional association with developmental delay and/or regression further worsens the prognosis.^12^, ^31^ Follow‐up data from our study group demonstrated that over 80% had global developmental delay and almost one‐third of infants died before the age of 10 years, with a significantly higher rate in the infantile spasms of known aetiology group*.* A quarter of our cohort developed refractory epilepsy and six were diagnosed with Lennox–Gastaut syndrome.

Since they were first described in 1841, infantile spasms have been extensively studied, yet the exact pathophysiological changes leading to spasm development remain obscure. Various animal models mimicking the pathogenic mechanisms of infantile spasms have been developed. Proposed mechanisms include stress‐induced hyper‐excitability, loss of inhibitory neurons, and significant damage to cortical and subcortical regions.^32^

Hypoxic brain injury in early life is a predisposing factor for infantile spasms. The pattern of injury on neuroimaging has been assessed to establish which infants are at greatest risk of developing infantile spasms. Gano et al.^33^ followed up a cohort of 176 infants with a history of HIE, eight of which developed infantile spasms. On comparing the neonatal MRI scans of infants with HIE who developed infantile spasms versus those who did not, basal ganglia/thalamic injury, along with brainstem and extensive cortical involvement, were highly associated with later development of spasms. Similarly, the neuroimaging results in our cohort of seven showed basal ganglia involvement in the majority (5 out of 7) and considerable bilateral cortical injury in the remainder.

Since 2009, evidence of the positive impact of therapeutic hypothermia on the mortality and developmental outcomes in HIE has been well substantiated.^34^ Therapeutic hypothermia was also proven to reduce the incidence of later epilepsy, but limited results are reported on its relation to infantile spasms.^35^ Among our HIE subset, direct correlation between spasm development and the severity of HIE (as per Sarnat staging) was demonstrated. The number of infants treated with therapeutic hypothermia who went on to develop spasms was appreciably lower than infants not treated with therapeutic hypothermia, yet the difference did not reach statistical significance.

With colocated paediatric and neonatal neurophysiology units, we had the opportunity to follow infants with a history of HIE who underwent neonatal EEG monitoring into their childhood years, with the ability to track changes on their EEG starting from the neonatal period through to the development of hypsarrhythmia and beyond, which makes this study unique.

The main limitation of this study was the cohort size. Since the cohort was small, the statistical tests had low power. Given the rarity of this disorder and relatively small number of cases, this is not surprising. In the group of infants with a history of HIE, only seven went on to develop infantile spasms; therefore, the effect of therapeutic hypothermia was difficult to ascertain. A much larger cohort is needed to prove/disprove a beneficial role of therapeutic hypothermia on later infantile spasms.

For the purpose of this study, we described the characteristics of infantile spasm cases and their outcome, with the emphasis on the degree of HIE injury and possible effects of therapeutic hypothermia in the HIE‐affected subgroup. Future plans are to look at the neonatal course in more depth, including a thorough evaluation and description of EEG recordings during the neonatal period, to help identify any baseline features predictive of the later development of infantile spasms.

Our study results indicate that, in the post‐therapeutic hypothermia era, HIE remains a leading cause of infantile spasms with an identified aetiology. We have determined that infants with severe HIE and severe EEG abnormalities in the neonatal period are at the greatest risk. We propose that larger, multicentre studies are required to examine the effect of therapeutic hypothermia on infantile spasm risk in this subgroup.

## Supporting information


**Figure S1:** Progression of electroencephalogram findings in a patient with history of hypoxic‐ischemic encephalopathy.
