# Supplementary material for: Impact of therapeutic hypothermia on infantile spasms: an observational cohort study
Source: Dev Med Child Neurol. 2019 Sep 13;62(1):62–8. doi: 10.1111/dmcn.14331 (PMC6916151; doi:10.1111/dmcn.14331)
Supplement: Supplementary file 1 — Figure S1: Progression of electroencephalogram findings in a patient with history of hypoxic‐ischemic encephalopathy. [file DMCN-62-62-s001.pdf]

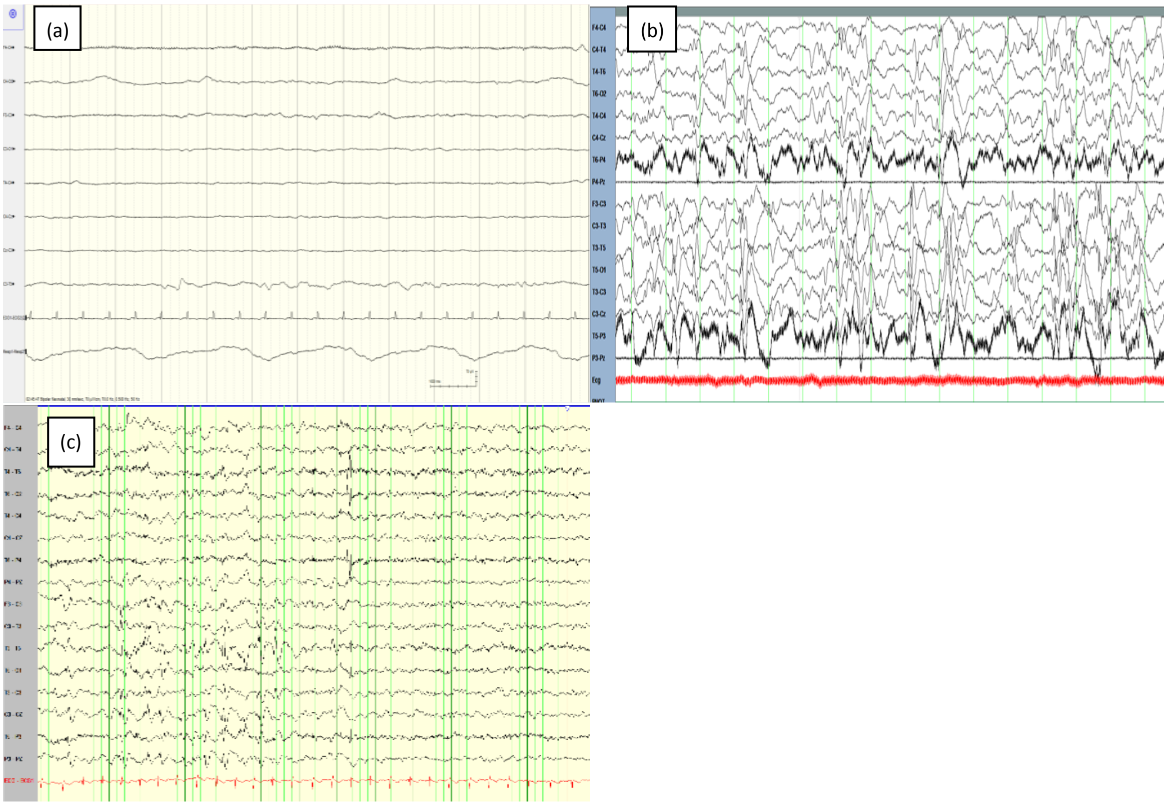

Figure S1: Progression of electroencephalogram (EEG) findings in a patient with history of hypoxic-ischemic encephalopathy.
(a) Neonatal EEG with severe electrographic encephalopathy. International 10–20 system lead position. Timescale: 30mm sec −1; sensitivity: 70uV cm−1.
(b) Hypsarrhythmic EEG at 6 months of age. Modified bipolar lead position. Timescale: 30mm sec−1; sensitivity: 15uV mm−1.
(c) EEG at 23 months, with features of Lennox–Gastaut syndrome. Modified bipolar lead position. Timescale: 30mm sec−1; sensitivity: 15uV mm−1.
